# MIF: Mood Improving/Inhibiting Factor?

**DOI:** 10.1186/1742-2094-11-11

**Published:** 2014-01-21

**Authors:** Joshua Bloom, Yousef Al-Abed

**Affiliations:** 1 Hofstra North Shore-LIJ School of Medicine, Hempstead, NY 11549, USA; 2 Center for Molecular Innovation, The Feinstein Institute for Medical Research, 350 Community Drive, Manhasset, NY 11030, USA

**Keywords:** MIF, Depression, Neuroinflammation, Neurogenesis, Antidepressant, Biomarker

## Abstract

Although major depressive disorder imposes a serious public health burden and affects nearly one in six individuals in developed countries over their lifetimes, there is still no consensus on its pathophysiology. Inflammation and cytokines have emerged as a promising new avenue in depression research, and, in particular, macrophage migration inhibitory factor (MIF) has been shown to be significant in depression physiology. In this review we summarize current research on MIF and depression. We highlight the arguments for MIF as a pro- and antidepressant species and discuss the potential implications for therapeutics.

## Introduction

Major depressive disorder (MDD) is a clinical syndrome defined by chronic disturbances in emotion and ideation that are accompanied by somatic or neurovegetative symptoms [1]. The disease has a worldwide lifetime prevalence of 12%, with the prevalence in developed countries (USA and Europe) as high as 18% [2]; this figure is increasing over time [3]. Additionally, depressive mood is often comorbid with other psychiatric conditions such as anxiety and eating disorders, as well as with chronic medical conditions such as cancer, cardiovascular disease, neurological disorders, and chronic inflammatory diseases [4]; this is often called ‘secondary depression’ [5]. Comorbid depression significantly worsens outcomes in coronary heart disease, diabetes mellitus, and stroke [6,7]. Depression can also cause cognitive symptoms [8] that can produce severe psychosocial deficits [9]. Despite these considerations, treatment for depression has not changed significantly in recent years. Current treatments do not adequately address cognitive deficits in depression [10], and there remain few solutions for treatment-resistant depression, which affects almost half of the patient population [11].

One of the reasons for the slow progress in this area is the lack of a unified theory of the pathobiology of depression. Several hypotheses are currently supported by research. One of the oldest is the monoamine theory, which asserts that depression is caused by a depletion of monoamines (such as serotonin or norepinephrine) in the brain [12]. Selective serotonin reuptake inhibitors (SSRIs) operate on this premise, and they are currently among the first-line treatments for major depression [13]. However, this theory fails to explain the delay in remission during treatment with SSRIs, or why depletion of monoamines does not reproduce depressive symptoms in healthy controls. As a result, a neurotrophic theory of depression has emerged: atrophic changes are found in the postmortem brains of MDD patients, and increases in neurogenesis or neuroplastic factors have antidepressant effects [12]. Any unified theory of depression would doubtless need to incorporate aspects of both of these hypotheses.

A large body of evidence has also pointed to an inflammatory etiology in depression [14]. Depressed mood develops in nearly a third of patients treated with recombinant interferon alpha, and is more prevalent in patients with chronic inflammatory diseases [15,16]. Systemic inflammation produces sickness behavior that resembles depression in both patients and rodent models [17]. One of the challenges of this hypothesis is explaining how peripheral cytokines can cross the blood brain barrier and affect the central nervous system to induce depression. One proposed explanation centers on the cytokine-activated enzyme indoleamine 2,3-dioxygenase, which has been shown to induce depression-like behavior. It degrades neural tryptophan into 3-hydroxykyurenin and quinolinic acid; in addition to being neurotoxic, these metabolites also drain local stores of tryptophan, which is a prerequisite in the synthesis of serotonin [18].

### Macrophage migration inhibitory factor

With mounting evidence for a role for cytokines in depression, macrophage migration inhibitory factor (MIF) has emerged as a strong candidate for a pathophysiological role. MIF is one of the first cytokines to be investigated, originally identified by its ability to prevent random migration of macrophages. It is released from intracellular pools by T- and B-lymphocytes, monocytes, macrophages, dendritic cells, neutrophils, eosinophils, mast cells, and basophils. It is also widely distributed in tissues [19]. Its release is triggered when cells are exposed to microbial products, pro-inflammatory cytokines, or specific antigens. Upon release, it acts in an autocrine and paracrine fashion to induce production of pro-inflammatory cytokines [20]. It also opposes the anti-inflammatory activity of glucocorticoids [21], which will be discussed later. Finally, it has been shown to have a role in cellular responses to DNA damage and cell cycle regulation [22]. MIF has been implicated in several disease conditions, including sepsis [23], acute respiratory distress syndrome [24], tuberculosis [25], and diabetes [26].

MIF has been shown to bind the transmembrane receptor CD74 and complex with CD44, with subsequent signal transduction via extracellular signal-regulated kinases (ERK1/ERK2), which are subtypes of mitogen-activated protein kinases (MAPK) [27]. This leads to several downstream effects that mediate the physiological effects of MIF. Particularly significant is the production of prostaglandin E2 (PGE2). Upregulation of phospholipase A2 (PLA2) is likely important in both the pro-inflammatory cascade and inhibition of glucocorticoid activity [28]. MIF also increases expression of TLR4, which is involved in immune responses to pathogenic bacteria as well as the pathogenesis of endotoxemia [29]. In addition, MIF promotes survival of pro-inflammatory cells by inhibition of the tumor suppressor p53 [30]. MIF has been shown bind and inhibit JUN-activation domain-binding protein 1 (Jab1), a coactivator of activator protein 1 (AP1), which is involved in cell growth. MIF also acts as an enzyme *in vitro*, showing D-dopachrome tautomerase and thiol protein oxidoreductase activities [31,32].

There are multiple lines of research pointing to a role for MIF in the pathobiology of depression. These findings have (i) identified MIF expression in the brain, particularly in areas significant to the behavioral symptoms of depression; (ii) established the significance of the hypothalamic-pituitary-adrenal (HPA) axis in depression, with which MIF has an intricate relationship; (iii) shown an interaction between MIF and both lifestyle and pharmacological antidepressant treatments; (iv) determined a connection between MIF and neurogenesis, another important avenue of depression research; and (v) explored MIF as a biomarker in major depression and other mood disorders. There is still much uncertainty about MIF’s exact pathophysiologic role, and whether its activity promotes or obstructs pathological processes in depression. The goal of this review is to summarize current research on the topic, highlight evidence for MIF as a pro- or antidepressant, and address the potential for future developments in this area.

### MIF in the central nervous system

Although MIF was originally identified as a product of T-lymphocytes, it was later discovered to be ubiquitous, with especially high expression rates in epithelia and endocrine tissues [20]. Several studies have also highlighted MIF expression in the central nervous system (CNS). Immunostaining of bovine brain has established MIF expression in the subependymal astrocytes, CA3/CA4 pyramidal cells of the hippocampus, and granule cells of the dentate gyrus [33]. Similar techniques have been used to localize MIF expression in rat brain to choroid plexus epithelia, ventricular ependymal cells, and cerebral astrocytes; the presence of MIF mRNA in astrocytes and neurons has also been confirmed with *in situ* hybridization [34]. An analysis of rat brain by Bacher and colleagues revealed MIF expression in neurons of the cortex, hypothalamus, hippocampus, cerebellum, and pons [35]. Conboy *et al*. used immunohistochemistry to establish MIF expression in astrocytes and the subgranular zone of the hippocampus [36]. Interestingly, several of these areas contain proliferating or maturing cell populations, which is an important consideration for neurogenesis. There is significant regional association with glucocorticoid activation.

MIF has also been isolated in human brain tissue, with high levels of MIF mRNA expression in all regions [37]. Human neural MIF maintains high expression levels throughout life (compared to other tissues, whose levels decline with age), which has led some to propose a maintenance role for MIF in isomerization of reactive catecholamine metabolites to neuromelanin precursors [38]. Neuromelanin has been shown to be neuroprotective in the pathobiology of Parkinson’s disease due to its role as a scavenger and sink for toxic metabolites [39]. MIF has putative roles in several CNS inflammatory conditions, including multiple sclerosis [40] and cerebral ischemia-reperfusion injury [41], as well as being implicated in tumor growth in the CNS [19].

Extensive research has been done in identifying brain areas significant in depression. Schmidt and colleagues identified corticostriatal projection neurons as being essential for antidepressant response [42]. A 2008 review of neuroimaging, lesioning, and postmortem analyses posits a visceromotor network underlying the physiology of emotion and mood, with dysfunction in this circuit leading to the symptoms of depression [43]. This network involves interplay between the medial prefrontal cortex, amygdala, hippocampus, and various limbic structures. It is noteworthy that this circuit includes the hippocampus, one of the areas previously identified as showing high MIF expression. This model has been applied to deep brain stimulation, an emerging approach to treatment-resistant depression [44].

### MIF and glucocorticoids

As mentioned above, MIF opposes the activity of glucocorticoids on the immune system. This activity of glucocorticoids is well understood, and oral corticosteroids are in use by 0.5% of the general population. Glucocorticoids are produced endogenously as cortisol and released by the adrenal glands upon stimulation by adrenocorticotropic hormone (ACTH), secreted by corticotropic cells of the anterior pituitary; those cells release ACTH in response to stimulation by hypothalamic corticotropin-releasing hormone (CRH). This process is called the HPA axis. Corticosteroids bind cytosolic receptors that dimerize and translocate to the nucleus, downregulating transcription of pro-inflammatory cytokines and decreasing production of prostaglandins [21]. These effects are mediated by interactions with NFkB [45], an important transcriptional regulator. Specifically, glucocorticoids upregulate expression of annexin 1 [46] and MAPK phosphatase 1 (MPK1) [47], which both cause downregulation of PLA2. In addition to being involved with the production of prostaglandins and leukotrienes from arachidonic acid, PLA2 also stimulates release of cytokines via Jun N-terminal kinases (JNK) [48].

As discussed above, MIF causes upregulation of PLA2, likely downstream of ERK1/2 signaling pathways [28]. It may also affect NFkB via its interaction with Jab1, which can lead to suppression of the inhibitory binding factor of NFkB (IkB) [22]. MIF is expressed in cells at every level of the HPA axis [49]. Its plasma levels follow a circadian rhythm that is comparable to that observed for plasma cortisol [21], and it is released from pituitary cells by CRH in a dose-dependent fashion [50]. Cortisol has also been shown to induce secretion of MIF with a bell-shaped dose response curve [51], in which high levels suppress MIF production. This seems to indicate a homeostatic balance between MIF and glucocorticoids, with the dominant species determining whether to promote immune responses (in infection) or dampen them (to protect from the harmful effects of inflammation).

It is well established that patients with MDD experience dysregulation of the HPA axis, manifesting as alterations in cortisol secretion and loss of suppression by dexamethasone [52,53]. These abnormalities manifest in 40 to 60% of patients with MDD [54]. This HPA dysregulation is similar to the hormonal changes observed in Cushing’s disease, albeit to a lesser degree [55]; interestingly, Cushing’s patients experience a greater incidence of mood disorders, which resolve upon normalization of cortisol levels [56]. Although patients with MDD do not experience Cushingoid symptoms *per se*, strong associations have been found between the hypercortisolism of depression and physical changes associated with Cushing’s disease, including hippocampal atrophy, cognitive impairment, and abdominal obesity [55]. These hormonal changes seem to be related to adrenal hyperresponsiveness to ACTH [54] as well as altered responses to glucocorticoids, especially at the level of negative feedback in the pituitary [17].

The relationship between altered glucocorticoid signaling and depression has been reiterated in mouse models, although there is still insufficient evidence to substantiate a true ‘glucocorticoid receptor theory of depression’ [57], especially since roughly half of MDD patients do not manifest HPA abnormalities. However, HPA dysregulation may represent one pathway among many that converge to produce the symptoms of depression. Significantly, stress and corticosteroids have also been shown to inhibit hippocampal neurogenesis; this effect is reversed by antidepressants [58]. MIF’s role in this scheme has been investigated: Edwards *et al*. found that MIF levels were 40% higher in healthy volunteers who showed depressive symptoms on the Beck Depression Inventory (BDI), and elevated MIF was associated with decreased cortisol response to acute stress and lower morning cortisol values [59].

### MIF and antidepressant treatments

Antidepressant response is a commonly used paradigm in depression research. Since the pathobiology and genetics of depression remain unknown, and many of its symptoms are impossible to replicate in an animal model, it has become necessary to design experimental models based on reproducible responses to established antidepressant treatments [60,61]. These models utilize both classical treatments, such as selective serotonin reuptake inhibitors (SSRIs) and electroconvulsive therapy (ECT), as well as new treatments like increased physical activity and deep brain stimulation [62].

MIF has been tested against traditional antidepressant treatments. An assay of motivated behavior has shown an association between the ERK1/2 pathway and responses to tricyclic antidepressants [63]. Conboy and colleagues determined that fluoxetine, a commonly administered SSRI [13], causes an increase in neurons immunoreactive for MIF. They also found that MIF knockout (KO) mice and mice given the MIF inhibitor [64] ISO-1 showed decreased neurogenesis after administration of fluoxetine. In addition, deletion of MIF resulted in increased depressive symptoms in the Porsolt forced swim test for behavioral despair (FST) and impairments in hippocampal spatial learning and memory in the Morris water maze. They concluded from these results that MIF is significant in the neurogenic effects of antidepressants [36].

Physical activity (PA) is emerging as an exciting new avenue of therapy for depression. PA has relatively few adverse effects, and can positively influence other physiological and psychological disorders. PA has been shown to be immunomodulatory, promoting expression of certain cytokines and immune cells and reducing others, as reviewed elsewhere [65]. PA also induces production of erythropoietin (EPO), a glycoprotein hormone involved in red blood cell hematopoiesis [66]. EPO and its receptor have recently been elaborated in the CNS [67], and they have been shown to have neurotrophic and neuroprotective effects [68].

Moon *et al*. found that the antidepressant effects of exercise were partially mediated by MIF, which they concluded functions as an antidepressant [69]. Using data from mRNA microarrays, they determined that both voluntary exercise and ECT induce MIF. Similar to the results from Conboy *et al*. [36], MIF^−/−^ mice showed increased depressive behavior and decreased antidepressant effects from exercise on the FST. Intracerebroventricular (icv) MIF administration had a direct antidepressant effect on the FST. They also analyzed gene expression patterns for brain-derived neurotrophic factor (BDNF), an important species in neurogenesis [70], and tryptophan hydroxylase-2 (Tph2), a rate-limiting enzyme in brain production of serotonin [71]. Both were upregulated in exercise and icv administration of MIF. Induction of Tph2 by MIF was matched with increased expression of serotonin in a recombinant cell line. It was further determined that these effects are all dependent on CD74 and ERK1/2, both established factors in MIF signal transduction [20,64].

### MIF and neurogenesis

MIF is known to have a role in embryonic development and cellular proliferation. As mentioned above, it promotes cell growth and inhibits apoptosis via inhibition of p53, a tumor suppressor protein. Swant *et al*. established in fibroblasts that RhoA GTPase is an important link between MIF and cyclin D1, which promotes cell cycle progression by phosphorylation of Rb, another tumor suppressor protein [72]. Inactivation of Rb leads to disinhibition of E2F, which promotes synthesis of S phase proteins and subsequent cellular proliferation [73]. MIF directly stimulates activation of RhoA.

Ito and colleagues determined that MIF is an important factor in embryonic development of zebrafish, a commonly used model for embryogenesis. Using whole-mount *in situ* hybridization (WISH) they detected widespread MIF expression in embryonic structures, including eyes, tectum, branchial arches, and gut structures. Using antisense Morpholino-mediated knockdown (MO), they determined that MIF MO fish displayed a reproducible phenotype of abnormal development in eyes and cartilage structures, and, significantly, in brain structures such as the tectum and fourth ventricle [74]. Similar expression patterns have been observed in avian, murine, and other mammalian models [75-77]. MIF has also been shown to promote proliferation and survival of neural stem progenitor cells *in vitro*[78].

It is notable that MIF expression in the brain is localized in regions of cellular proliferation. As discussed above, MIF expression has been found in proliferating cells of the subgranular zone of the hippocampus, and is modulated by treatments affecting neurogenesis (chronic stress, corticosteroids, and antidepressants). MIF deletion by genetics or inhibitors also attenuates both basal and induced neurogenesis [36]. Similarly, Moon *et al*. found that MIF induces the production of BDNF [69], whose role in neurogenesis is well-established [70]. A summary of results from significant animal studies of MIF and depression can be found in Table 1.

**Table 1:** Animal studies of macrophage migration inhibitory factor (MIF) in the setting of depression or depressive etiologies

**Authors**	**Model (n)**	**Intervention**	**Analysis**	**Results**
Conboy *et al*. [36]	Adult male Wistar rats (48)	Chronic unpredictable stress, chronic corticosterone	Immunohistochemistry for Ki-67, MIF-IR cells in dentate gyrus	MIF co-localizes with proliferative markers
				MIF levels correlate with neurogenesis
	WT and MIF KO mice (16)	Fluoxetine (ip, od/14days), ISO1 (od/14days)	Immunohistochemistry for PCNA, DCX, BrdU, Ki-67, MIF-IR cells in dentate gyrus	Loss of MIF results in decreased basal and antidepressant-stimulated neurogenesis
	WT and MIF KO mice (9, 16, 20, 8)	Acute stress exposure	ELISA for serum corticosterone, Western blot for receptor expression	MIF KO mice show no significant difference from WT in levels of serum glucocorticoids or receptor expression
	WT and MIF KO mice (52, 20)	None	FST, water maze, acoustic fear conditioning	MIF KO mice show increased behavioral despair
				MIF KO mice show impaired hippocampal spatial learning and memory, intact amygdalar fear conditioning
Moon *et al*. [69]	Male Sprague–Dawley rats (13, 4)	Voluntary exercise (running wheel), ECS (55 mA pulses, 100/s)	RT-PCR, Western blot, immunohistochemistry for MIF mRNA/protein	MIF mRNA is upregulated by exercise and ECS
	Neuro-2A and RBL-2H3 cells (3); WT and MIF KO mice (12 to 16); male Sprague–Dawley rats (8 to 12)	MIF (300 ng/mL), exercise/ECS (see above), MIF (icv), CD74 siRNA, CT04 (5ug/mL), U0126 (10 uM)	RT-PCR for candidate genes (Bdnf, Fgf2) and neurogenesis genes (Dcx, Pax6), HPLC for 5HT, Western blot for *P*-ERK1/2	MIF induces expression of BDNF and Tph2, and also increases intracellular concentrations of 5HT
				Effects on BDNF, Tph2, and 5HT are CD74 and ERK1/2 dependent
	WT and MIF KO mice (12 to 16); male Sprague–Dawley rats (16 to 20)	Exercise (see above), MIF (icv)	FST	MIF KO mice show diminished antidepressant effects of exercise
				Exogenous recombinant MIF has antidepressant activity

ECS = electroconvulsive shock; FST = forced swim test; icv = intracerebroventricular injection; ip = intraperitoneal injection; IR = immunoreactive; KO = knockout; od = once daily; WT = wild-type.

### MIF as a biomarker

With inflammation implicated in pathophysiology of depression, several groups have examined cytokines as depression biomarkers [79]. Rodent studies have indicated that serum MIF increases in response to acute stress [49,80]. Several groups have examined MIF levels in human serum in the context of depressive symptoms. A study of male undergraduate students presented with a public speaking task determined that subjects with mild to moderate depression on the BDI demonstrated higher baseline serum levels of MIF as well as increased lymphocytes [59,81]. Similar results have been reported in pregnant women, where an association was determined between depressive symptoms and increased MIF. Increased serum MIF was also observed after an immune challenge in pregnant patients with depressive symptoms as measured by the Center for Epidemiologic Studies Depression Scale (CES-D) [82]. Interestingly, studies of healthy patients with negative mood symptoms as measured by the Zung self-rating depression scale (SDS) showed no significant association between serum MIF and SDS mood scores [83]; associations were found with IL-1β, a species known to be elevated in depression [84].

MIF has also been examined as a biomarker in the context of clinical depression. In a drug trial examining celecoxib add-on therapy to reboxetine (a norepinephrine reuptake inhibitor), MDD patients had an increased serum MIF at baseline with no change during treatment [85]. Similar results were found in an analysis of leukocyte mRNA expression in serum from participants in the Genome-based Therapeutic Drugs for Depression (GENDEP) project. In addition to observing increased MIF in MDD patients compared to healthy controls, the group also found that treatment responders had significantly higher levels of serum MIF than patients who resisted treatment. MIF levels were shown to reduce over time in this study, but this was not associated with treatment response [86]. Results of human studies with MIF and depression are summarized in Table 2.

**Table 2:** Controlled studies of macrophage migration inhibitory factor (MIF) in major depressive disorder (MDD) or depressive mood

**Authors**	**Subjects**	**Depression measures**	**Intervention(duration)**	**Analysis (t)**	**Pertinent results**
Hawkley *et al*. [81]	75 subjects	BDI	Public speaking stress task (once)	Serum MIF (0, 15 minutes)	MIF levels are increased in subjects showing mild to moderate depression (BDI)
					MIF levels are unaffected by the public speaking stress task
Edwards *et al*. [59]	126 healthy subjects	BDI	Public speaking stress task (once)	ELISA for serum MIF (0, 3, 15, 45 minutes)	MIF levels are increased at baseline in subjects showing high depressive symptoms (BDI)
		UCLA-R			
					MIF levels do not change over the time course measured
Christian *et al*. [82]	22 pregnant subjects	CES-D	Vaccination for influenza virus (once)	ELISA for serum MIF (0, 1 weeks)	Pregnant women with depressive symptoms (CES-D) show increased MIF levels at 1 week
Katsuura *et al*. [83]	209 healthy subjects	Zung-SDS	None	Multiplex suspension array for serum levels of multiple immune mediators (0)	MIF levels are not significantly associated with depressive symptoms (SDS)
Musil *et al*. [85]	32 MDD patients, 20 healthy controls	*DSM-IV*	Treatment with reboxetine and add-on celecoxib (5weeks)	ELISA for serum MIF, TGFB, and sCD14 (0, 3, 5 weeks)	MIF levels are increased at baseline in MDD patients
		HRSD			
					MIF levels are unchanged during reboxetine treatment
					Celecoxib reduces HamD scores but does not affect MIF levels
Cattaneo *et al*. [86]	74 MDD patients, 34 healthy controls	*DSM-IV*	Treatment with escitalopram or nortryptiline (8 weeks)	qPCR for serum leukocyte mRNA levels of several candidate genes (0, 8 weeks)	MIF mRNA levels are increased at baseline in treatment-responsive MDD patients; MIF mRNA levels decrease during treatment, but with no correlation to treatment response
		MADRS			
		HRSD			
		BDI			

All studies collected serum from subjects at the times indicated (0 = baseline) for measurements of peripheral MIF levels. BDI = Beck Depression Inventory; CES-D = Center for Epidemiologic Studies Depression Scale; *DSM-IV* = *Diagnostics and Statistics Manual of Mental Disorders, 4th edition*; HRSD = Hamilton Depression Scale 17-item version; MADRS = Montgomery-Asberg Depression Rating Scale; SDS = Self-Rating Depression Scale; UCLA-R = Revised UCLA Loneliness Scale.

## Discussion

**Figure 1:**
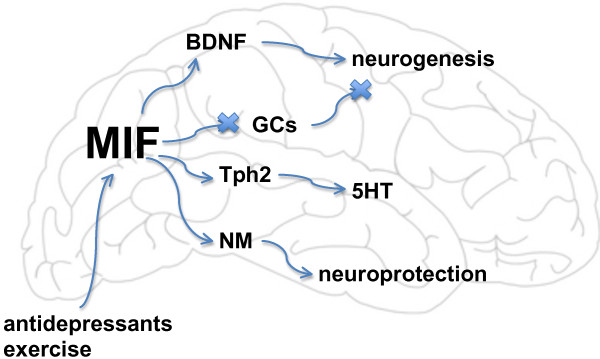
**Putative roles for macrophage migration inhibitory factor (MIF) in depression.** GCs = glucocorticoids; NM = neuromelanin. (Single column fitting figure; color for Web only).

Despite significant evidence for MIF involvement in the pathobiology of depression, some uncertainty remains about its exact role. MIF is an established species in the brain with suggested protective roles against neurodegenerative disease [38]. Multiple groups have identified a role for MIF in mediating antidepressant activities, and have shown that loss of MIF results in an antidepressant phenotype; there is even evidence that MIF has direct antidepressant effects. These studies have linked MIF to monoamine production and neurogenesis, both implicated in the pathobiology of depression [36,69]. Conversely, increased serum MIF has been identified in both patients with major depression and healthy subjects with depressive symptoms [81,82,85,86], although these studies have shown mixed results [83]. At least one study has associated these changes with the HPA axis, which has also been implicated in depression [59]. See Figure 1 for a summary of putative roles for MIF in depression.

It seems counterintuitive to assert that MIF is both an antidepressant and a biomarker of depression. However, it is important to realize that the Conboy and Moon studies were working with MIF native to the brain, while the biomarker studies were analyzing MIF levels in peripheral blood. MIF does not cross the blood brain barrier [87,88], and differential expression in the two areas may explain the differing observations. MIF levels in plasma may be incidental to the mechanisms of depression or may arise as a consequence of a different but related process. It is also possible that the two results are not mutually exclusive, and increased MIF in depressed individuals is a physiological adaptation to the pathobiological changes of depression. It is notable in this regard that increased MIF in depressed patients has been found to correlate with treatment response [86].

MIF underlies the pathophysiology of several disease conditions, and MIF inhibition is well characterized and widely used in research [64]. Anti-MIF antibodies are currently being investigated in Phase I clinical trials [89]. It seems inevitable that some form of MIF inhibitors will soon become available at the clinical level. When this occurs, MIF’s role in depression - whatever it may be - will be highly relevant. If MIF is found to promote depression, then MIF inhibitors could be investigated as antidepressants; ISO-1, the most tested MIF inhibitor, has already been shown to cross the blood brain barrier [36]. If MIF acts as an antidepressant, then anti-MIF therapeutics can be engineered not to cross the blood brain barrier, bypassing depression as a possible off-target effect.

## Conclusion

There are clear gaps in the research concerning MIF and depression. Future studies should work to elucidate the relationship between central and peripheral MIF in depression, if any exists. Further work should also be done to clarify MIF’s role as a pro- or antidepressant and its place in the pathobiology of depression. It may be useful to further analyze relationships with factors in and out of the monoamine and neurogenic pathways that have been shown to impact depression. Imaging studies have emerged as an important modality in neuropsychiatric disorders, including depression [90,91]; it may prove interesting to test how alterations in MIF expression affect the presentation of the disease on imaging studies. One advantage in these projects will be the fact that MIF is a well-studied molecule, with both established inhibitors and KO strains in rodent models.

Although there is much to be done, it seems beyond doubt that MIF has great potential in studies of the mechanisms of major depression. In addition to interacting with known elements involved in the physiological changes of depression, it is also active in the brain and can be shown to have independent effects on the depression phenotype. In addition to expanding our knowledge about this still-enigmatic disease, such studies can also inform current drug development efforts with anti-MIF therapy, as well as possibly provide the stagnant field of antidepressant treatment with a valuable new target in modifying the course of this disease.

## Abbreviations

ACTH: adrenocorticotropic hormone; AP1: activator protein 1; BDI: Beck Depression Inventory; BDNF: brain-derived neurotrophic factor; CES-D: Center for Epidemiologic Studies Depression Scale; CNS: central nervous system; CRH: corticotrophin releasing hormone; ECT: electroconvulsive therapy; EPO: erythropoietin; ERK: extracellular signal-related kinase; FST: Porsolt forced swim test; GENDEP: Genome-based Therapeutic Drugs for Depression project; HPA: hypothalamic-pituitary-adrenal; HRSD: Hamilton Depression Scale 17-item version; icv: intracerebroventricular; IkB: inhibitory binding factor of NFkB; IFN: interferon; IL: interleukin; Jab1: JUN-activation domain binding protein 1; JNK: JUN N-terminal kinase; KO: knockout; MADRS: Montgomery-Asberg Depression Rating Scale; MAPK: mitogen-activated protein kinase; MDD: major depressive disorder; MIF: macrophage migration inhibitory factor; MO: Morpholino-mediated knockdown; NF: nuclear factor; PA: physical activity; PGE2: prostaglandin E2; PLA2: phospholipase A2; SDS: Zung self-rating depression scale; SSRI: selective serotonin reuptake inhibitor; Tph2: tryptophan hydroxylase-2; UCLA-R: Revised UCLA Loneliness Scale; WISH: whole-mount *in situ* hybridization.

## Competing interests

Joshua Bloom has received an MD/PhD candidate stipend from the Hofstra North Shore LIJ School of Medicine. Yousef Al Abed is the inventor or co-inventor of several small-molecule inhibitors of MIF. The authors declare no other conflicts of interests.

## Authors’ contributions

JB designed and drafted the manuscript; YA conceived of the study, helped draft the manuscript, and gave final approval of the version to be published. Both authors read and approved the final manuscript.
